# Advanced neuroimaging and criminal interrogation in lie detection

**DOI:** 10.1515/med-2024-1032

**Published:** 2024-09-05

**Authors:** Valentina Opancina, Vladimir Sebek, Vladimir Janjic

**Affiliations:** Department of Radiology, Faculty of Medical Sciences, University of Kragujevac, Kragujevac, Serbia; University Clinical Center Kragujevac, Kragujevac, Serbia; Department of Criminalistics, Faculty of law, University of Kragujevac, Kragujevac, Serbia; Regional police directorate of Kragujevac, Republic of Serbia, Police Directorate, Ministry of interior, Kragujevac, Serbia; Department of Communication Skills, Ethics and Psychology, Faculty of Medical Sciences, University of Kragujevac, Kragujevac, Serbia

**Keywords:** neuroimaging, functional imaging, lie detection, information, criminal interrogation

## Abstract

Hidden information is the key to many security issues. If there is a reliable method to determine whether someone withholds information, many issues of this type can be resolved. However, until now, no method has proven to be reliable, but technical discoveries in the field of neuroimaging have caused a surge of new research in this area. Many neuroimaging techniques can be used, but functional magnetic resonance is the newest method, and its use in extracting and evaluating information from subjects could be the most significant, given that it records brain states in parallel with current mental activity/behavior, enabling the establishment of correlational links between them. Because the brain state displayed during fMRI imaging is the dependent variable measured during stimulus/task condition manipulation, it is necessary to use fMRI data in combination with complementary criminal interrogation techniques to gather information. This could be particularly important when standard interrogational techniques are not enough in order to preserve the common good, especially in “ticking bomb” situations. In this study, we review aspects of the possibility of utilizing advanced neuroimaging in combination with criminal interrogation in cases of serious criminal acts that threaten public safety.

## 1 Introduction

Hidden information is the key to many security issues. If there is a reliable method to determine whether someone withholds information, many issues of this type can be resolved. However, until now, no method has proven to be reliable, but the technical discoveries in the field of imaging technologies of the brain, the so-called neuroimaging, have caused a surge of new research in this area. In addition, ethical questions regarding the application of this technology for investigative interviewing have also been raised [1]. Combining a universal model of investigative interviews with advanced neuroimaging technologies could “open the door to the future” of criminal interrogation.

The development of brain imaging techniques and brain activity has progressed over time and continues to evolve. Besides their use in the clinical environment, the possibility of their utilization in the context of national security has emerged since September 11, 2001 [2]. Neuroimaging technologies have been presented as a potentially valuable asset in events of threats to security, stability, and peace, where standard criminal interrogation techniques to counter terrorism may not be sufficient [3]. Different neuroimaging techniques can be used to evaluate suspects or criminal offenders depending on the main goal [4]. Functional magnetic resonance imaging (fMRI) is the newest method, and its use in extracting and evaluating information from subjects could be the most significant, given that it records brain states in parallel with current mental activity and/or behavior, enabling the establishment of correlational links between them. However, the brain state displayed during fMRI imaging is the dependent variable measured during stimulus/task manipulation [5]. Therefore, it is necessary to correlate fMRI data with complementary interrogation techniques, and use them combined for gathering information, which would be particularly important in order to preserve the common good fight against terrorism, as well as in “ticking bomb” situations since the use of fMRI in everyday criminal practice would be too time-consuming and could be ethically debatable.

Because of the above, we conducted a review of the current literature in order to present advanced neuroimaging modalities and their possible use with criminal interrogation techniques in order to extract necessary information and detect lies as a valuable asset to fight serious criminal acts that threaten public safety.

## 2 Criminal interrogation techniques

Many techniques have been used to extract information from disinclined individuals, such as interrogation, polygraphs, and torture [5].

In everyday criminal practice, investigative interviews are used in order to obtain information from witnesses, victims, and suspects. Because of this, it earns an important place in most police investigations, in which both theorists and practitioners agree. Therefore, it is crucial for the examiner to receive accurate and reliable information so that the result is a successful administration of justice. Withholding valuable information during debriefing can damage or interrupt investigations. Therefore, the skill of conducting investigative interviews is learned and practiced, and models such as the PEACE model are used [6,7].

The PEACE model was designed as a framework for investigative interviewing in any situation and with any type of respondent, making it universal. The fact that it was developed by the police in England and Wales does not reduce its importance due to some specificities of a legal nature, and this led to its spread to the practices of other countries all over the world. What is also significant and should be emphasized is that the PEACE model has been in use for more than two decades, which speaks in favor of the positive results of this way of conducting interviews in criminal investigations. The acronym PEACE stands for five different stages in the interviewing process as follows: (1) planning and preparation, (2) engaging and explaining, (3) giving a statement, clarification and dispute (account, clarification, and challenge), (4) closing the conversation (closure), and (5) evaluation [6].

Apart from the PEACE model, there are other interrogation techniques used in criminalistics, such as the Reid method and the Kinesic interview [7,8]. The kinesic interview focuses mainly on nonverbal communication. The Reid method is mainly used in the USA by law enforcement and security professionals. It contains three elements: factual analysis (evaluation of the suspect’s individual characteristics, such as socio-demographic facts, and motivation for the crime), interviewing (including standardized investigative questions and questions with the aim of provoking behavioral symptoms of truth or deception), and interrogation (including nine steps: “(1) positive confrontation, (2) theme development, (3) handling denials, (4) overcoming objections, (5) procurement and retention of suspect’s attention, (6) handling the suspect’s passive mood (7) presenting an alternative question, (8) having the suspect orally relate various details of the offense, and (9) converting an oral confession to a written confession”) [7,8].

As part of criminal processing, as an auxiliary technical tool, the polygraph can be used with the aim of quickly and relatively reliably eliminating innocent persons from the circle of suspects in order to concentrate the operative activity on the likely perpetrator, if possible, to obtain a confession from the perpetrator, and to find material evidence in formation. The application of the polygraph is based on a connection that exists between the psychological survival of a certain conditioned situation, for example, by asking a specific question and remembering a certain event for which respondents are bound by strong emotions. By activating intellectual content that is strongly emotionally colored (such as fear of exposure due to committed crime actions, awareness of something bad and forbidden has been done, guilty conscience), violent physiological processes in the body are provoked, for example, accelerated heart rate, blood pressure changes, breathing rate changes, activation of endocrine glands, increased sweating, facial redness, and expansion or narrowing of the pupils. These devices register automatic and self-regulating physiological changes in the body, which are not under the control of the subject’s consciousness and will and are conditioned by the emotional survival of a conflict situation. The reliability of the polygraph is high, although the possibilities should not be overestimated. The accuracy of polygraph test results is largely dependent on the abilities and knowledge of the polygraph examiner. Certain categories of criminals do not respond to polygraph testing, such as callous psychopaths, given that they do not emotionally survive the crime they committed. Certain categories of patients with mental disorders, and persons under the influence of narcotics, alcohol, and certain medications are not suitable for polygraph testing [9]. Also, research studies show that polygraphs still have high false-positive and false-negative rates because they cannot be used as a single lie detection technique [10].

Also, there are situations in which the standard interrogational techniques are insufficient. We encounter situations in which national security and safety are in question because of terrorist attacks. In crises such as this, when states are faced with imminent disasters, timely and concrete action is necessary in order to remediate the consequences and prevent the occurrence of new ones, which requires the application of all available resources in order to obtain true information and prevent catastrophes. Such conditions are known in the literature after the terrorist attack in the USA in 2001, after which states allowed the use of torturous methods known as “enhanced interrogation techniques” under “ticking time bomb” exception [2,11,12]. Nevertheless, since severe stress (imminent to occur during enhanced interrogation) impairs cognitive functions that disrupt the process of retrieving information and detecting deceit, neuroimaging methods may present a better method for this use [13].

Therefore, new techniques are needed in the field of criminal interrogation, as well as the complementary use of standard criminal interrogation techniques with functional neuroimaging tools. This is especially important in cases where suspects may be highly trained and indoctrinated terrorists and criminals who work in large organized criminal groups where standardized interrogational techniques may not be sufficient.

## 3 Neuroimaging

It is possible to classify these neuroimaging techniques as structural or functional. Structural techniques demonstrate the brain anatomy and potential pathologic entities, including computed tomography (CT) and magnetic resonance imaging (MRI). Functional techniques show brain function and include electroencephalography (EEG), positron emission tomography (PET), single-photon emission computed tomography (SPECT) fMRI imaging, and functional near-infrared spectroscopy (fNIRS) [14].

Specific categories of brain imaging are used for specific purposes in clinical practice. CT scan is currently the first line of brain diagnostics and is used primarily in acute states, such as trauma and hemorrhage, but also as the initial scan in the process of lesion identification. Being a more sensitive technique than CT, the use of MRI contributes to the more precise delineation of pathological entities, analysis of brain lesions (such as inflammatory lesions, demyelination, vascular lesions, and tumors), and neurosurgical/neurointerventional planning [15]. Given the fact that MRI does not use ionizing radiation, such as CT, monitoring of various brain conditions is recommended. PET and SPECT are techniques used in the field of nuclear medicine that contribute to metabolic and functional data. When combined with CT and MRI, these neuroimaging techniques provide intricate anatomical and metabolic information, which is especially valuable for oncological diseases and dementias [15,16].

### 3.1 Electroencephalography

EEG is an electrophysiological technique that estimates dynamic cerebral function owing to its extraordinary temporal sensitivity. Its principle is based on the creation of an electrogram, which records spontaneous electrical activity arising from the brain [17]. It is a noninvasive technique, and electrodes are traditionally placed on the scalp surface [18,19]. It is possible to have electrodes implanted intracranially; this type of EEG is called deep EEG. Also, electrodes used during the process can be noninvasive (with the aim of collecting EEG signals) or invasive and are being surgically implanted but show better signal acquisition, higher spatial resolution, and more detailed findings [20,21]. In general, EEG detects brain signals that depict the postsynaptic potentials of pyramidal neurons located in the neocortex and the allocortex. The neural activity recorded by EEG is the sum of the excitatory and inhibitory postsynaptic potentials of the rather large neuronal group that fires synchronously [17,18,19,20,21].

EEG is mainly used by neurologists in order to measure brain electrical activity for the diagnosis and monitoring of different neurological disorders, such as epilepsy, sleep disorder research, detection of drug effects, and monitoring of the depth of anesthesia during surgical procedures [15]. Technological developments have created feedback devices that can convert brain signals measured by EEG into interpretable data. This would allow “human–computer interaction” and possibly identify various emotional states, cognitive status, and neurological disorders. Further development of this technique is needed, as well as serious research studies to demonstrate the specificity and sensitivity of the technology [17].

### 3.2 fMRI

Functional magnetic resonance imaging utilizes a strong magnetic field to create images of biological tissues.” Various tissue characteristics can be obtained, as well as the distinction between tissue types, depending on the pulse sequence used. It is important to note that different pulse sequences are used for fMRI depending on the collection of structural or functional data [5]. fMRI is a magnetic resonance imaging method that registers “changes in the regional blood volume and flow that are associated with cognitive activity” [15]. It works on the principle of recording differences in MR signals from oxyhemoglobin and deoxyhemoglobin, because active brain areas consume more oxygen than inactive areas [5]. For the brain field/area to become active, cognitive tasks need to occur, after which local microvasculature is employed. Arterial blood flow increases and consequently increases the utilization of oxygen, or better, oxyhemoglobin. The rapid change in the blood oxygenation levels (after 1–2 s) after neuronal activity provokes a change in the MR signal, which is known as “blood oxygenation level-dependent” (BOLD) signal [5]. These changes and BOLD signaling allow scientists to confine brain activity on a timely basis (second by second) and within millimeters of the source [22].

In addition to being classified as a noninvasive imaging technique, fMRI is also called a correlational neuroimaging technique since it establishes correlational links (not causal) between brain states parallel with behaviors or ongoing mental processes. In fMRI studies, the outcome or dependent variable is the brain state, which has been recorded during the manipulation of the cognitive task/stimulus [5]. Moreover, it is important to note that none of the changes in cerebral blood oxygenation are caused intentionally and are physiological, which puts fMRI in the group of noninvasive imaging methods [23].

There are limitations to the use of fMRI. This requires the use of a magnetic field, which may be contraindicated in specific situations [24]. Also, it measures only the oxyhemoglobin to deoxyhemoglobin ratio without separate detection of each. Finally, it does not measure neuronal activity directly; it only measures blood oxygenation changes, which may be hindered by different vasoactive processes [25].

### 3.3 Functional near-infrared spectroscopy

Functional near-infrared spectroscopy is a noninvasive, portable technique that is cost-effective and safe for patients with contraindications for MRI. FNIRS is an optical imaging technique that measures changes in the light absorption of various hemoglobin species [26]. This is possible because the tissue has relative transparency to light for infrared wavelengths (650–925 nm range). Oxyhemoglobin and deoxyhemoglobin absorb light more efficiently than other tissues, whereas deoxyhemoglobin absorbs better below 790 nm and oxyhemoglobin above 790 nm. FNIRS identifies the changes in various light-absorbing molecule concentrations and, in that sense, makes it possible to analyze brain energy metabolism. Similar to fmRI, it can measure alterations in oxygenated and deoxygenated hemoglobin and detect changes in neuronal activation. However, unlike fMRI, it measures oxyhemoglobin and deoxyhemoglobin separately [25].

However, the biggest limitation of FNIRS is its low spatial and temporal resolutions. Engineers are working on new technical developments to improve it, creating multichannel NIRS and new signal processing methods in order to allow FNIRS to be widely used as a clinical tool. However, their low accuracy and precision still present obstacles [25]. The temporal resolution of FNIRS is even less optimal than that of EEG, which makes this technique below the standard for the detection of individual neurological disorders. Also, spatial resolution, which is of great interest in cognitive tests and functional localization, is less than that in fMRI and is generally suboptimal [25,27]. Another important limitation of FNIRS is the lack of a cortical region approach, such as the ventral part of the frontal cortex, basal ganglia, or a large part of the cerebellum [25]. However, this method has great potential, and its complementary use with other techniques may be of great assistance in brain function analysis.

### 3.4 Neuroimaging and interrogation

Criminalistics is usually defined as a science that studies, finds, and perfects scientific and practical experience-based methods and means, which are the most suitable to discover and clarify the criminal act, discover and bring the perpetrator to justice, secure and fix all the evidence to establish the (objective) truth and prevent the execution of planned and unplanned criminal acts. In other words, it is the science of techniques, tactics, and methods of operational, investigative, and other judicial actions, as well as crime prevention [9]. Over time, scientific contributions to intelligence agencies have increased, and the place of medical expertise in the interrogation process has become well-established. The aid of physicians is described in the Philippines war, when US interrogators, during the administration of the “water cure”, asked doctors for assistance who ordered the addition of salt in order to cause irritation of the nasal mucosa and speed up the interrogation process. During the Cold War, psychiatrists and psychologists helped CIA agents in the interview process by helping them to use psychological stressors. In the 21st century, reports indicate the use of psychotropic drugs during interviews with criminal and terrorist suspects [28]. Recently, it was found that a more sophisticated approach could be used for special interrogations with the help of neuroimaging.

Currently, research data indicate that the most advanced neuroimaging technology being discussed as an interrogation tool is fMRI. The literature suggests that studies published at the beginning of the 21st century draw much interest in fMRI use in lie detection [29]. fMRI study indicated that “the anterior cingulate cortex and superior frontal gyrus are components of the basic neural circuitry of deception” [30]. Similar studies have been undertaken in order to prove these results, which were thought to be of great use, especially in counterterrorism interrogations [28].

It was reported that after September 11, intelligence operatives collaborated with physicians and that different neuroimaging technologies have been used, such as CT, MRI, and EEG, along with fMRI studies. They indicated the use of EEG in order to detect massive brain electrical activity when specific keywords were being said (in English and the native language of assumed terrorists) during the scanning. The technique was developed by Mossad and London University College [31]. Also, the literature reports the creation of an EEG-based system called brain fingerprinting, which can be used to discover knowledge of information or its absence in crime-related situations. It measures event-related brain potentials and also uses the Concealed Information Test in order to discover whether an individual recognizes information from real life, such as crime. Several studies have evaluated its accuracy, but the latest one has shown that it is not yet an optimal tool for criminal investigation. However, it has great potential, especially for confirmation of accuracy in the innocence claim of the suspect and for detecting knowledge in a perpetrator [32].

Clinical trials have been performed in order to evaluate fMRI and polygraphs for lie detection. The Concealed Information Test was used in this prospective study, which included consecutive and counterbalanced interrogations of subjects using fMRI and polygraph. The results showed that fMRI experts were 24% more likely to discover concealed information than polygraph experts [33]. These data justify the need to further develop fMRI in the field of criminal interrogation.

Different methods show that neuroimaging tools, especially fMRI, are used not only for lie detection but also for gathering criminal intelligence, such as the recognition of audio or visual stimuli or verbal data, such as the name of a person or city [28]. Still, we need to consider that many of these findings are protected data, and we hope to have more published literature on this topic in the future.

### 3.5 fMRI use in lie detection

fMRI in the process of interrogation and subsequent lie detection has two potential uses: assessing familiarity and detecting deception based on the neural representation of memory and conflict, respectively. When discussing interrogation, assessing familiarity is necessary for the criminal investigation process, such as asking if the suspect has been at the crime scene. Instead of gathering information through the PEACE model, fMRI could be employed in special cases, and in that case, the criminal interrogator could show the picture of the crime scene to the suspect while the suspect is being scanned by functional MRI. The BOLD signal map can show a pattern that indicates prior experience with the crime scene location. Another possibility for fMRI use is the detection of deception based on the neural detection of conflict between truth and lie. The conflict happens when someone is lying because the truth is the natural answer (basic assumption of different techniques for lie detection), and in order for someone to lie, he/she must inhibit the truth that creates the conflict [34].

The use of fMRI in lie detection studies is based on the detection of BOLD signals and their evaluation as a consequence of the contrast between two or more actions. BOLD signal maps of lying participants are compared to those when they are telling the truth, which allows the creation of patterns and conclusions about the neural correlates between lying and nonlying states [5]. This is possible only within the same study design for lying and nonlying conditions (case and control) since any difference, such as different stimuli, may interfere with the map and, consequently, with the pattern [35]. In addition, researchers have created algorithms for data patterns and the distinction between lie and truth using fMRI [5,36].

Lie detection via functional MRI and BOLD signals has also been investigated under laboratory conditions using a mock sabotage script and deception testing. The study included healthy adults, under no influence of any medication, who were screened and then randomly divided into “mock-crime” and “no-crime” groups. The first group was asked to perform a mock criminal act (stealing CDs), while the second group was asked to restrain the same act. Participants were then scanned using fMRI while being interviewed about mock crimes. The same two groups of participants also underwent fMRI deception tests, in which they were asked to steal either a watch or a ring and then, later on, to report that they had not stolen anything while being scanned under fMRI. In the deception test, researchers were able to detect a lie in 69.45% of the participants, which formed the validation group. When scoring mock crime, results have shown 100% sensitivity and 33% specificity, which in laboratory conditions showed high sensitivity but low specificity for fMRI use in lie detection testing [37].

Different studies have used fMRI in order to analyze the neurocognitive basis of legal judgments in criminal law cases, which included only female participants who committed crimes with good intentions. fMRI findings have shown that the right temporoparietal junction has a special position in the state of the mind process of criminal perpetrators. Also, it was shown that the right dorsal lateral prefrontal cortex influences the resolution of moral conflicts [38].

When reviewing the published literature, it was found that activation of certain brain regions was specific for truth: “Brodmann area 40, the striatum, left thalamus, the superior parietal lobe” [39]; while activation of other brain regions presented similar maps for truth and deceit: “cerebellum, posterior cingulate gyrus, prefrontal cortex, and the precuneus”. It was also indicated that deception may implicate inhibition of answers that are truthful by the fact that most of the brain activity happens in the prefrontal cortex bilaterally, which is connected to “response inhibition” [39]. Depending on the type of lying, spontaneous or well-rehearsed, different regions are activated. Spontaneous lies activate “the precuneus, anterior cingulate, the ventrolateral prefrontal cortex, the dorsolateral prefrontal cortex, and posterior visual cortex anterior prefrontal cortex”, while rehearsed lies activate “the right anterior prefrontal cortex, Brodmann Area 10, and the precuneus” [39]. It was displayed that there was no activation of the limbic system in lying [40].

However, fMRI is not yet standardized for use in the field of criminalistics and needs to be better adapted and undergo further testing. Also, there is a need to put the use of fMRI in criminal cases into the legal framework, which must be done at national legislative levels. Ethical questions also need to be addressed. However, it should be noted that fMRI has great potential for use in the criminal interrogation of suspects profiled as terrorists or criminals in large organized criminal groups. fMRI may be of great assistance to criminalistics experts as a complementary tool to standardized interrogative techniques, such as the PEACE model and polygraph.

### 3.6 Transcranial stimulation in the detection of lie deception

Neuroscience developments have introduced the use of noninvasive brain stimulation tools in order to test conclusions from neuroimaging findings and to determine if the neuroimaging implication is causally connected to behavior such as deception or to validate neuroimaging results linked to deception. For this purpose, two noninvasive techniques are used: transcranial magnetic stimulation (TMS) and transcranial direct current stimulation (tDCS) [41]. TMS discharges brief current pulses that quickly change the magnetic field around the coil held on the head. This causes current flow in the cortical regions, which focally stimulates neurons and modulates their activity. tDCS uses anode and cathode electrodes on the scalp and modulates the excitability of the cortex in this way. Anodal tDCS boosts cortical excitability in the targeted brain area, while cathodal tDCS has the opposite effect. Studies have concluded that excitability changes occur as a result of subthreshold neuronal membrane depolarization [43]. However, more research is needed on this topic to obtain more reliable data, compare it, and provide new information on the possibility of using transcranial stimulation in the detection of lies.

### 3.7 Application of neuroimaging in criminal law

To date, there has been no proven method for the truth assessment of legal testimony, but the potential utilization of fMRI in this matter has been discussed. fMRI evidence has been applied in several legal cases in the US courts [42]. However, after researching the cases in all three of them, the evidence was not admitted due to the strict jurisdiction standards on scientific evidence admissibility (testability of the technique, peer-review publications, error rate, standards, and general acceptance of the method by the scientific community) [42]. However, the acceptance of fMRI as legal evidence depends on the jurisdictional system of a country. Nevertheless, private companies have continued to advertise private fMRI scans to test lies (for example, “No Lie MRI” and “CEPHOS”).

Research has shown that there is potential for using fMRI as a superior method to lie detector - recognition of brain regions implicated in telling truth, lies, and false memories, which are especially problematic for validation of witness testimony [43]. Nonetheless, intentional lie detection is the most interesting for experts by fMRI, and the study results suggest that in this case lies activate the dorsolateral prefrontal cortex, and in the case of false memories, the right anterior hippocampus is activated. However, it must be taken into account that during scanning and interview, activation of these regions may occur due to something unrelated since these brain regions have executive control functions [44].

**Figure 1:**
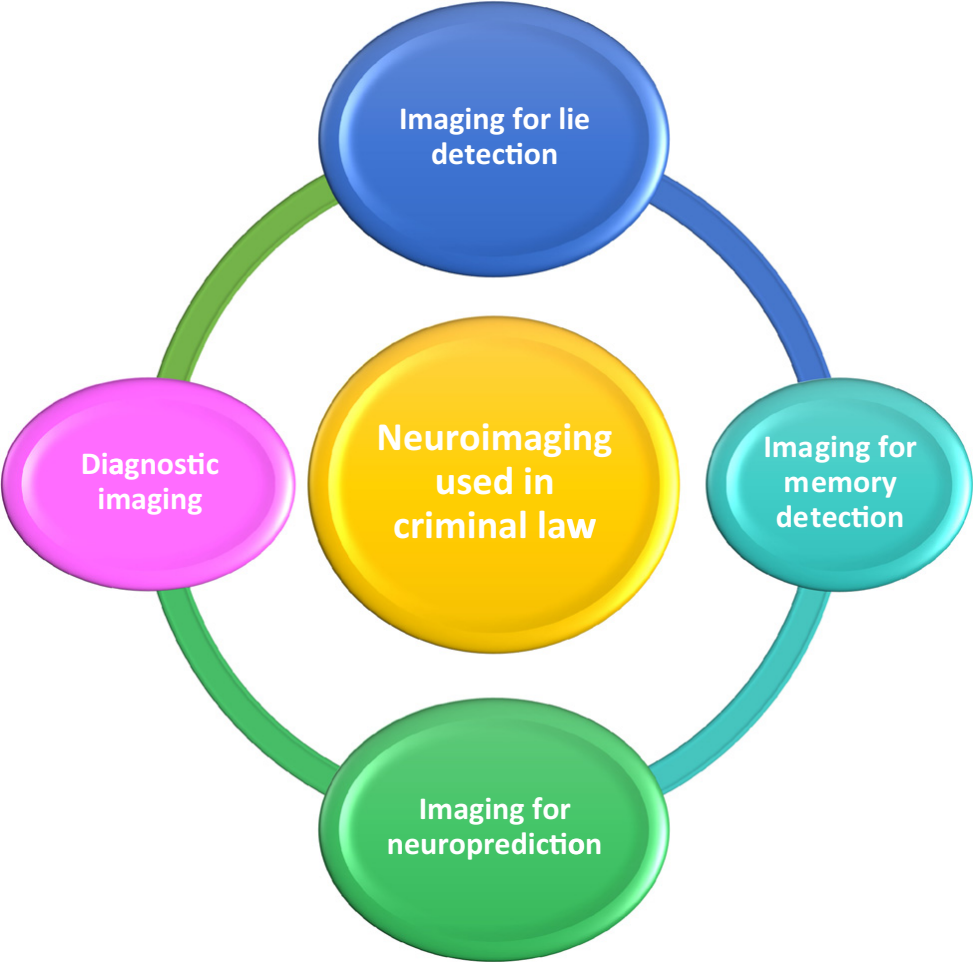
Classification of neuroimaging techniques for use in criminal law.

The literature has suggested the classification of neuroimaging, which contributes to criminal law in four groups, as presented in Figure 1 [45,46].

The application of neuroimaging modalities in criminal law is miscellaneous, from the discovery of guilt to legal responsibility, risk of recidivism, and fitness to stand trial [46]. A novel application is the combination of artificial intelligence with standard neuroimaging modalities for use in criminal law – “mind reading” or detection of “real-time thoughts”. This technology was used in a laboratory setting, and the results showed high accuracy, with 91% identification of suicidal thoughts [47]. This is still in the experimental phase, but the results and possible applications show us that we may open new doors in the world of forensic imaging and the use of neuroimaging in legal proceedings. Before the new technology arrives in court, scientists must work on the limitations of neuroimaging in these cases, such as possible manipulation (recalling emotional memories during scanning or moving the tongue) [45]. Subject motions during fMRI present the biggest limitation of scanning because even a movement of a few millimeters can compromise the results [46].

Neuroimaging can be used in situations where physical or legal coercion is used on the suspect [48]. Physical coercion may be used in order to keep the suspect still during brain scanning. A different possibility is pharmacological assistance in order to prevent suspicion of moving during examination [48]. Legal coercion does not involve physical force but threatens the suspect with negative consequences for the sake of her decision to participate in the examination. This may be considered necessary because some neuroimaging (such as fMRI) demands active involvement of the subject during examination [49]. During the examination, the subject may be asked to move their fingers, press buttons, answer questions, and observe presented stimuli, such as pictures or voices [45]. Physical coercion, in this case, is useless, and special attention must be paid to countermeasures that the subject (study participant or suspect) may use. It is necessary to note that physical coercion interferes with right-to-body integrity, which is guaranteed by the European Convention of Human Rights (ECHR), Article 8 [46]. The participant needs to decide whether they will undergo neuroimaging and sign an informed consent form. However, there are circumstances in criminal law where free and informed consent may not always be guaranteed. Interferences may be justified and essential in order to protect national security, prevent crime, protect rights, freedoms, health, or morals, and secure the economic well-being and public safety of the country [46].

### 3.8 Application of neuroimaging in criminal interrogation

When discussing the validity of advanced neuroimaging modalities in criminal interrogation, we must take into account that much of the results are laboratory and experimental phases, especially those that involve “mind-reading”. Therefore, differences between study groups that included healthy individuals randomly selected from the community and groups of criminal offenders or suspects for criminal acts must be assessed in order to diminish possible countermeasures by participants.

It is also known that a high percentage of criminal offenders meet some psychopathy criteria; in this condition, deception should be expected. fMRI studies have investigated a group of subjects who committed crimes and were diagnosed with psychopathy. All of the participants had anti-social personality disorder, and a large percentage did not have the expected fMRI study results–BOLD response patterns in the prefrontal region during instructed deception [50].

The use of structural neuroimaging in criminal investigations may be necessary in order to confirm mental disorders, a range of psychopathological entities, advanced age, and some personal traits such as high anxiety [50]. Diagnostic neuroimaging might also be necessary before functional neuroimaging in order to gain information on brain anatomy and possible pathologies before fMRI testing. This might be necessary in order to draw correct conclusions from fMRI studies because different cognitive, personality, or brain pathology factors might interfere with the fMRI validity for lie detection.

The accuracy of fMRI studies on lie detection varies but increases to 90% [51]. It should be considered that there are limitations in terms of individual fMRI recordings, as well as confusion and stress that this method would cause in subjects. In addition, the stillness of the participants can be a major issue, as even small movements can create artifacts and interfere with results [51]. Also, there are patients with contraindications for MRI, such as claustrophobia and implants from ferromagnetic materials, who could not undergo this type of examination. Apart from medical conditions, possible criminal suspects or offenders might be juveniles, addicts, or mentally unstable, and in those terms, unfit to undergo functional MRI; moreover, we must note that technology is expensive, it is not widely available, and this type of testing would require highly trained professionals, both from the medical field and criminalistics.

However, the applicability of neuroimaging has not yet been standardized for use in the field of criminalistics, and it needs to be better adapted and further tested. There is also a need to put the use of neuroimaging, such as fMRI in criminal cases, into the legal frame, which must be done at national legislative levels. However, it should be considered that fMRI has great potential for use in the criminal interrogation of suspects profiled as terrorists or criminals in large organized criminal groups. fMRI may be of great assistance to criminalistics experts as a complementary tool to standardized interrogative techniques, such as the PEACE model and polygraph.

In order to discuss the application of neuroimaging in criminal investigations, in addition to imaging characteristics and study results, we must also take into account legal and ethical aspects. Until now, there have been no ECHR cases concerning the use of coercive brain imaging in criminal justice. Therefore, it is necessary to compare the possible utilization of neuroimaging in these cases with standard methods used in criminal investigations [46].

DNA analysis and fingerprinting have been used in criminal investigations. Their use consists of personal data utilization, which is unique to each individual [46]. Similarly, the anatomy and activity of the brain contain unique personal data since not even monozygotic twins have identical brain anatomy, which has been confirmed in the literature [52]. Therefore, the nature, use, and context of neuroimaging in brain activity detection during criminal interrogation might be assessed similarly to the use of DNA and fingerprints in order to discover offenders or future perpetrators, to link a suspect to the crime, and to present the data about someone’s involvement in the crime.

Finally, it should be noted that the question of neuroimaging, crime, and lie detection presents complex cases with not only scientific but also ethical and political challenges. Further research and public debates are needed in order to achieve the beneficial use of neuroimaging in criminal investigations and to avoid potentially harmful purposes. “Mind-reading” and lie detection in combination with artificial intelligence may open the door for many problems, such as invasion of individual privacy, confidentiality issues, predictive testing of any nature, legal discrimination, commercial use, etc.

## 4 Future directions

Future directions should manage the legal use of advanced neuroimaging, especially fMRI, in criminal investigations with the aim of data gathering. In addition, it is necessary to have more publicly funded studies since the benefit of this type of examination is enormous, and there should not be a conflict of interest between private companies and researchers. We encourage more studies in this field, especially with the aim of distinguishing intentional lies from forgotten information.

## 5 Conclusion

In this article, we review aspects of the utilization of advanced neuroimaging in criminal interrogation, with the main focus on the use of fMRI in cases of serious criminal acts that threaten public safety, such as terrorist attacks. This field is still being developed and researched in laboratory settings in many countries, and its possible use in cases that deal with safety and national well-being demands the scientific community to work on these limitations. Advanced neuroimaging can be used in security situations, especially as a counterterrorism measure, since it is more ethical and reliable than torture, but more upgrading is needed in this area in order to increase the validity of this method in data extraction from individuals. We must keep in mind that the polygraph has been used in criminal interrogation for a century and still raises controversial questions. Therefore, it is expected that this topic has contentious issues that can be resolved over time, but we need to pay attention to the positive effects that advanced neuroimaging may have when used in criminalistics, improve the techniques and standardize them, in order to outweigh the negative issues and limitations.

## Abbreviations

BOLD: blood oxygenation level-dependent
CT: computed tomography
ECHR: European Convention of Human Rights
EEG: electroencephalography
fMRI: functional magnetic resonance
fNIRS: functional near-infrared spectroscopy
MRI: magnetic resonance imaging
PET: positron emission tomography
SPECT: single-photon emission computed tomography
tDCS: transcranial direct current stimulation
TMS: transcranial magnetic stimulation
